# Polymorphisms of *pfcrt*, *pfmdr1*, and *K13-propeller* genes in imported falciparum malaria isolates from Africa in Guizhou province, China

**DOI:** 10.1186/s12879-020-05228-8

**Published:** 2020-07-16

**Authors:** Danya She, Zhengyan Wang, Qiuguo Liang, Lidan Lu, Yuting Huang, Ke Zhang, Dong An, Jiahong Wu

**Affiliations:** 1 Key Laboratory of Environmental Pollution Monitoring and Disease Control, Ministry of Education, Guizhou Medical University; Department of Parasitology; Provincial Key Laboratory of Modern Pathogen Biology, Guizhou Medical University, Guiyang, 550025 China; 2 Guizhou Provincial Center for Disease Control and Prevention, Guiyang, 550004 China

**Keywords:** *Plasmodium falciparum*, Antimalarial drugs, Resistance, Polymorphism, Haplotype

## Abstract

**Background:**

Imported falciparum malaria from Africa has become a key public health challenge in Guizhou Province since 2012. Understanding the polymorphisms of molecular markers of drug resistance can guide selection of antimalarial drugs for the treatment of malaria. This study was aimed to analyze the polymorphisms of *pfcrt*, *pfmdr1*, and *K13*-*propeller* among imported falciparum malaria cases in Guizhou Province, China.

**Method:**

Fifty-five imported falciparum malaria cases in Guizhou Province during 2012–2016 were included in this study. Their demographic information and filter paper blood samples were collected. Genomic DNA of *Plasmodium falciparum* was extracted from the blood samples, and polymorphisms of *pfcrt*, *pfmdr1*, and *K13*-*propeller* were analyzed with nested PCR amplification followed by sequencing. Data were analyzed with the SPSS17.0 software.

**Results:**

The prevalence of *pfcrt* K76T, *pfmdr1* N86Y, and *pfmdr1* Y184F mutation was 56.6, 22.2, and 72.2%, respectively, in imported falciparum malaria cases in Guizhou Province. We detected two mutant haplotypes of *pfcrt,* IET and MNT, with IET being more commonly found (54.7%), and five mutant haplotypes of *pfmdr1*, of which NFD was the most frequent (53.7%). There were totally 10 combined haplotypes of *pfcrt* and *pfmdr1*, of which the haplotype IETNFD possessed a predominance of 28.8%. In addition, three nonsynonymous mutations (S459T, C469F, and V692L) and two synonymous mutations (R471R and V589V) were detected in *K13*-*propeller*, all having prevalence less than 6.0%. In particular, a candidate K13 resistance mutation, C469F, was identified for the first time from Democratic Republic of the Congo with the prevalence of 2.0%.

**Conclusions:**

The high prevalence of IET haplotype of *pfcrt* and NFD haplotype of *pfmdr1* suggests the presence of chloroquine, artemether/lumefantrine, and dihydroartemisinin/piperaquine resistance in these cases. Therefore cautions should be made to artemisinin therapy for *P. falciparum* in Africa. Continuous monitoring of anti-malarial drug efficacy in imported malaria cases is helpful for optimizing antimalarial drug therapy in Guizhou Province, China.

## Background

Malaria remains an important public health problem in tropical and sub-tropical countries. It has been estimated that there were 216 million clinical cases of malaria and approximately 445,000 deaths in 2016, of which nearly 92% occurred in Africa, according to the World Health Organization (WHO) data [1]. In 2010, the Chinese government launched ‘an action plan for malaria elimination’ in the aim to eliminate malaria throughout the country by the end of 2020. Since then, great progress has been made to reduce the morbidity and mortality of malaria [2]. Although there has been no indigenous malaria case in the past endemic areas, the imported cases have become a big public health challenge in China. In 2016, a total of 3317 imported cases were reported in China, accounting for 99.9% of all [3]. In addition, most of the imported cases were infected with *Plasmodium falciparum* (*P. falciparum*).

Artemisinin-based combination therapy (ACT) has been recommended for the treatment of uncomplicated falciparum malaria in nearly all areas as recommended by WHO, since the emergence and spread of multi-drug resistant *P. falciparum* strains [4]. The ACT combines a fast-acting but rapidly-cleared artemisinin derivative with a long-lasting partner drug. Artesunate/amodiaquine (AS-AQ) and artemether/lumefantrine (AL) are the first-line drugs for uncomplicated *P. falciparum* malaria in Africa. In China, AS-AQ and dihydroartemisinin/piperaquine (DHA-PPQ) are recommended for uncomplicated *P. falciparum* infection [5]. The ACT therapy indeed shows a high efficacy, but an emerging ACT resistance has raised serious concern. Since the first report of emergence of *P. falciparum* resistance to artemisinin in 2008 in western Cambodia [6], such resistance has spread to all countries of the Greater Mekong Sub-region [7–9]. The presence of resistance to ACT partner drugs has caused treatment failure with DHA-PPQ, AL, etc. [10–12]. Therefore, it is important and necessary to survey the emergence and the distribution of artemisinin and partner drug resistance in order to guide public health measures and rational administration at the level of local government.

It has been widely accepted that *pfcrt, pfmdr1*, and *K13-propeller* are candidate genes for resistance to anti-malarial drugs [10, 13, 14]. Single nucleotide polymorphisms (SNPs) have been found in exon 2 of the *pfcrt* gene at codons 74, 75, and 76, which are associated with resistance of *P. falciparum* to chloroquine (CQ) and amodiaquine (AQ) [15, 16]. Among them, the K76T mutation is the primary mediator of CQ and AQ resistance, which is always accompanied by M74I and N75E mutations to maintain adequate fitness of the K76T resistant isolates [10, 14]. Studies on *pfmdr1* gene have identified 5 resistance-associated SNPs, of which the N86Y and Y184F are more common to Asian- and African-derived parasites while S1034C, N1042D, and D1246Y are found more frequently in South America [17]. Experimental evidence indicates that the combination of 86Y/184Y/1246Y which creates the ‘YYY’ haplotype contributes to the recrudescence and reinfections following AS-AQ or AQ treatment [18, 19]. The NFD haplotype (86 N/184F/1246D) reduces parasite susceptibility to AL, and can be selected by treatment with AL [18, 19]. Other mutations (S1034C and N1042D) are primarily associated with altered sensitivity to lumefantrine (LMF), mefloquine (MQ), and artemisinin [20].

Clinical artemisinin resistance, which manifests as the delayed parasite clearance, was first reported in Pailin, Western Cambodia in 2009 [6, 21]. This resistance is primarily modulated by mutations in the propeller domain of *P. falciparum* chromosome 13 (*K13*-*propeller*) in Southeast Asia, including 5 SNPs (N458Y, Y493H, R539T, I543T, and C580Y) verified so far [22, 23]. The C580Y SNP has also been reported in Africa [22]. Molecular surveillance of *K13-propeller* polymorphism has been recommended as a complementary tool to evaluate the presence of artemisinin resistance in malaria pandemic countries.

Currently, imported malaria is not only a threat to public health but also has become the most serious challenge for Chinese government to eliminate malaria within years [24]. There have been many studies on imported malaria cases from different areas conducted in China [25–27], but with most of the cases being from Africa, they provided inconsistent results on the drug-resistance associated gene polymorphisms and their frequencies. In this study, we set out to evaluate polymorphisms of *pfcrt*, *pfmdr1*, and *K13-propeller* genes in imported falciparum malaria cases from Africa, in order to provide guidance on antimalarial drug administration for the local government of Guizhou Province.

## Method

### Data collection

Malaria case data were provided by Guizhou Provincial Centre for Disease Control and Prevention. Fifty-five patients who returned from Africa during 2012–2016, diagnosed with malaria initially by microscopy of Giemsa’s solution-stained thick and thin blood smears at local hospitals or local County Centres for Disease Control and Prevention, and confirmed at Guizhou Provincial Center for Disease Control and Prevention by microscopic examination and nested PCR, were included in this study [28]. Blood filter papers from each patient were prepared and stored in individual plastic bags at − 20 °C after being air dried for DNA extraction. Malaria parasite species identification was carried out by nested PCR as previously described [28]. All the 55 malaria cases were confirmed to be infected with *P. falciparum* without mixed infections. The imported cases were from 15 African countries and were roughly categorized into the West Africa (*n* = 41, including Angola, Benin, Cameroon, Republic of the Congo, Democratic Republic of the Congo, Equatorial Guinea, Gabon, Ghana, Liberia, Nigeria, the Republic of Guinea, and Zambia) and the East Africa (*n* = 14, Mozambique, Tanzania, and Uganda) .

### SNP analysis

Parasite genomic DNA was extracted from the filter paper blood using a QIAamp DNA mini kit (Qiagen, Valencia, CA, USA) following the manufacturer’s instructions. We assessed *P. falciparum* polymorphisms at alleles of *pfcrt* M74I, N75E, K76T; *pfmdr1* N86Y, Y184F, S1034C, N0142D, D1246Y, and the mutation of PF3D7_1343700 kelch propeller domain gene (PF13_0238, also called *K13-propeller*), a molecular marker of artemisinin resistance, using nested PCR amplification followed by sequencing. The primer sequences were all from published literature [27, 29, 30] and are shown in Table 1.

**Table 1:** Primer sequences in this study

Gene	Locus	Round	Primers	Sequence (5′ → 3′)	Amplicons (bp)
*pfcrt* [27]	74,75,76	1st	CRT1–1	CCGTTAATAATAAATACACGCAG	537
			CRT1–2	CGGATGTTACAAAACTATAGTTACC	
		2nd	CRT2–1	TGTGCTCATGTGTTTAAACTT	145
			CRT2–2	CAAAACTATAGTTACCAATTTTG	
*pfmdr1* [29]	86,184	1st	MDR1–1F	TTAAATGTTTACCTGCACAACATAGAAAATT	612
			MDR1–1R	CTCCACAATAACTTGCAACAGTTCTTA	
		2nd	MDR1–2F	TGTATGTGCTGTATTATCAGGA	526
			MDR1–2R	CTCTTCTATAATGGACATGGTA	
	1034, 1042	1st	1042-A	GTCGAAAAGACTATGAAACGTAGA	350
			1042-C	CTCAAATGATAATTTTGCAT	
		2nd	1042-B	GATCCAAGTTTTTTAATACA	
			1042-C	CTCAAATGATAATTTTGCAT	
	1246	1st	1246-A	GTGGAAAATCAACTTTTATGA	410
			1246-B	TTAGGTTCTCTTAATAATGCT	
		2nd	1246-C	GACTTGAAAAATGATCACATT	
			1246-D	GTCCACCTGATATGCTTTT	
*K13*-*propeller* [30]		1st	K13–1-1	CGGAGTGACCAAATCTGGGA	2063
			K13–1-2	GGGAATCTGGTGGTAACAGC	
		2nd	K13–2-1	GCCTTGTTGAAAGAAGCAGA	849
			K13–2-2	GCCAAGCTGCCATTCATTTG	

PCR was performed based on previous publications with minor modification [27, 29, 30]. Briefly, for the 1st round of PCR, 2.0 μL genomic DNA template, 12.5 μL Premix rTaq, 1.0 μL forward primer (10 μM), 1.0 μL reverse primer (10 μM), and ddH_2_O (up to 25.0 μL) were mixed and subjected to the following program: initial denaturation at 95 °C for 3 min, followed by 35 cycles of 95 °C for 30 s, 52 °C for 30 s, and 72 °C for 1 min, and a final extension at 60 °C for 5 min. For the 2^nd^round of PCR, 2.0 μL products from the 1st round of PCR with 25.0 μL Premix rTaq, 1.0 μL forward primer (10 μM), 1.0 μL reverse primer (10 μM), and ddH_2_O (up to 50.0 μL) were mixed and subjected to the following program: initial denaturation at 95 °C for 3 min, followed by 35 cycles of 95 °C for 30 s, 54 °C for 30 s, and 72 °C for 60 s, and a final extension at 72 °C for 5 min. Five microliters of products from the 2nd round of PCR were analyzed by 1.2% agarose gel electrophoresis. The major bands were harvested and purified for bi-directional DNA sequencing (Sangon Biotech, Shanghai, China).

### Data analysis

Sequence alignment and analysis were carried out using DNAstar 7.0 software (DNASTAR Inc., Madison, WI, USA). The nucleotide and amino-acid sequences of *pfcrt*, *pfmdr1*, and *K13-propeller* from *P. falciparum* 3D7 strain (Genbank ID: Pf3D7_0523000, Pf3D7_0709000, Pf3D7_1343700) were used as the reference for alignment. Data were imported into Excel to construct database, and were analyzed with Person’s Chi square test or Fisher exact test using the SPSS 17.0 software. *P* < 0.05 was considered as statistically significant.

## Results

### Demographics of patients

A total of 55 filter paper blood samples were collected from imported falciparum malaria cases who returned from 15 African countries to Guizhou Province during 2012–2016, with a male/female ratio of 12.75:1 (51:4) and mean age of 37.6 ± 9.31 years old (range: 25–59). In addition,74.55% (*n* = 41) of the imported cases were from West Africa, and 25.45% (*n* = 14) of them were from East Africa (See Table 2).

**Table 2:** Geographic distribution of the imported falciparum malaria cases from Africa in Guizhou Province during 2012–2016

geographical division	country name	cases
West Africa	Angola	4
	Benin	1
	Cameroon	3
	Democratic Republic of the Congo	4
	Equatorial Guinea	1
	Gabon	1
	Ghana	1
	Liberia	14
	Nigeria	6
	Republic of the Congo	2
	the Republic of Guinea	3
	Zambia	1
East Africa	Mozambique	1
	Uganda	11
	Tanzania	2

### SNPs and haplotypes of the pfcrt gene

PCR products (145-bp) of the *pfcrt* gene covering codons 74–76 were sequenced in 96.4% (53/55) of the cases. The prevalence of K76T mutation was 56.6% (30/53), and the other two mutations N75E and M74I had a total prevalence of 54.7% (29/53). The proportion of *pfcrt* haplotype IET was significantly different between cases from East Africa and West Africa, while the MNT proportion did not differ significantly. The detail information of the mutations is shown in Table 3 and Fig. S1.

**Table 3:** Prevalence of SNPs and mutation haplotypes of *pfcrt* gene

SNPs / mutation haplotypes	Number of isolates and total prevalence (*N* = 53) n (%)	95% CI	Number of isolates from East Africa (*N* = 13) n (%)	Number of isolates from West Africa (*N* = 40) n (%)	*P*-value
K76T	30 (56.6)	(0.4269, 0.6938)	3 (23.0)	27 (67.5)	0.005
N75E	29 (54.7)	(0.4141, 0.6805)	3 (23.0)	26 (65.0)	0.008
M74I	29 (54.7)	(0.4141, 0.6805)	3 (23.0)	26 (65.0)	0.008
IET	29 (54.7)	(0.4141, 0.6805)	3 (23.0)	26 (65.0)	0.008
MNT	1 (1.9)	(−0.0178, 0.0555)	0	1 (2.5)	0.57

### SNPs and haplotypes of the pfmdr1 gene

*pfmdr1* genotyping was successfully performed in 98.2% (54/55) of cases for codons 86, 184, 1034, 1042, and 1246. No mutations were identified at codons 1034 and 1042. The 184F mutation was the most-frequent non-synonymous mutation (72.2%, 39/54)) among all the mutations, comprising a prevalence of 85.7 and 62.5% in cases from East Africa and West Africa, respectively, with no significant difference between them (*P* = 0.407). The 86Y mutation was detected in 14.3% of East Africa and 25% of West Africa isolates, with an overall prevalence of 22.2%. The 1246Y mutation was not detected in East Africa, but in 7.5% of West Africa isolates, with a total prevalence of 5.6%. There were 5 mutation haplotypes for *pfmdr1*, including NFD, YFD, YFY, YYD, and NYY, with prevalence of 53.7, 14.8, 3.7, 3.7, and 1.9%, respectively. None of the 5 haplotypes had significant difference between East Africa and West Africa. Detailed information of the mutations is shown in Table 4, Fig. S2, and Fig. S3.

**Table 4:** Prevalence of SNPs and haplotypes of *pfmdr1* gene

SNPs / mutation haplotypes	Number of isolates (*N* = 54) n (%)	95% CI	Number of isolates from East Africa (*N* = 14) n (%)	Number of isolates from West Africa (*N* = 40) n (%)	*P*-value
N86Y	12 (22.2)	(0.111, 0.333)	2 (14.3)	10 (25.0)	0.407
Y184F	39 (72.2)	(0.603, 0.841)	13 (92.9)	26 (65.0)	0.107
D1246Y	3 (5.6)	(−0.005, 0.117)	0	3 (7.5)	0.292
NFD	29 (53.7)	(0.404, 0.669)	11 (78.6)	18 (45.0)	0.226
YFD	8 (14.8)	(0.050, 0.238)	2 (14.3)	6 (15.0)	0.948
YFY	2 (3.7)	(−0.013, 0.087)	0	2 (5.0)	0.394
YYD	2 (3.7)	(−0.013, 0.087)	0	2 (5.0)	0.394
NYY	1 (1.9)	(0.001, 0.037)	0	1 (2.5)	0.55

### Combined haplotypes of pfcrt and pfmdr1

Both *pfcrt* gene and *pfmdr1* gene were genotyped successfully in 52 cases and the combined haplotypes are summarized in Table 5. They had a total of 10 combined haplotypes, predominated by IETNFD and MNKNFD with prevalence of 28.8 and 26.9%, respectively.

**Table 5:** Prevalence of correlated *pfmdr1-pfcrt* haplotypes (*N* = 52)

*Pfcrt*			*pfmdr1*			Number of isolates (%)	95% CI
74	75	76	86	184	1246		
M	N	K	N	Y	D	6 (11.5)	(0.028548, 0.202222)
M	N	K	N	F	D	14 (26.9)	(0.148670, 0.389792)
M	N	K	Y	Y	D	1 (1.9)	(−0.0180973, 0.056559)
M	N	K	Y	F	D	2 (3.8)	(−0.013808, 0.090732)
I	E	T	N	Y	D	5 (9.6)	(0.016026, 0.176282)
I	E	T	N	F	D	15 (28.8)	(0.165322, 0.411602)
I	E	T	Y	F	D	5 (9.6)	(0.016026, 0.176282)
I	E	T	Y	F	Y	2 (3.8)	(−0.013808, 0.090732)
I	E	T	N	Y	Y	1 (1.9)	(− 0.0180973, 0.056559)
M	N	T	N	Y	D	1 (1.9)	(−0.0180973, 0.056559)

### SNPs of K13-propeller

**Fig. 1:**
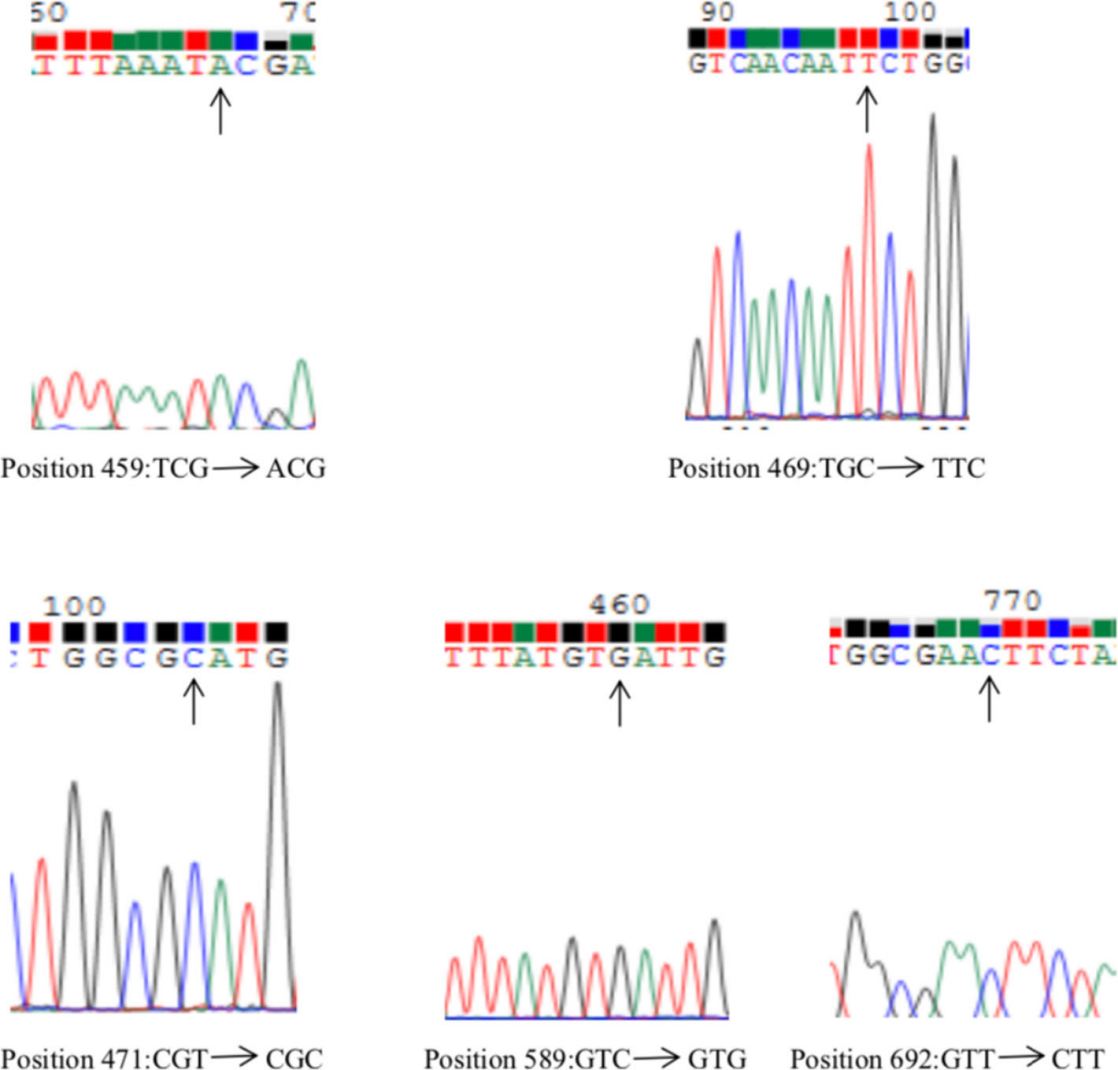
chromatograms of sequence analysis on mutation position of the *K13*-*propeller* gene in *Plasmodium falciparum* isolates imported from Africa in Guizhou Province. The arrow shows the mutation position

To investigate the *K13*-*propeller* SNP, an 849-bp PCR product was successfully sequenced in 90.9% (50/55) of the samples. Five SNPs were observed in 7 samples, including 3 nonsynonymous mutations and 2 synonymous mutations (Table 6 and Fig. 1). The three nonsynonymous mutations were S459T, C469F, and V692L, with equal prevalence of 2.0% (*n* = 1). The two synonymous mutations were R471R and V589V, with prevalence of 6.0% (*n* = 3) and 2.0% (*n* = 1), respectively.

**Table 6:** Polymorphisms observed in the *K13*-*propeller* in *P. falciparum* isolates

Codon position	Amino acid reference	Nucleotide reference	Amino acid mutation	Nucleotide mutation	Number of isolates (%)	Original country
459	S	TCG	T	ACG	1 (2)	Tanzania
469	C	TGC	F	TTC	1 (2)	Democratic Republic of the Congo
471	R	CGT	R	CGC	3 (6)	Angola (2), Gabon (1)
589	V	GTC	V	GTG	1 (2)	Benin
692	V	GTT	L	CTT	1 (2)	Liberia
696	A	GCC	V	GTC	1 (2)	Nigeria

## Discussion

Guizhou Province is located in the southwest of China and was once one of the provinces with greatest malaria burden. No indigenous cases have been reported since 2012 after a long-term strategy on malaria control in Guizhou [28]. However, imported malaria cases, especially falciparum malaria, have become the biggest risk that hinders malaria control and elimination in Guizhou.

In this study, we investigated the drug resistance-associated mutations of *P. falciparum* from imported cases from Africa in Guizhou Province during 2012–2016. We found that the prevalence of K76T mutation (56.6%) was highest among the three mutations of *pfcrt*. The K76T mutation has been confirmed as a key marker of CQ resistance by epidemiological, reverse genetic, and heterologous expression studies [31, 32], therefore CQ is not preferred for treatment of falciparum malaria cases with *pfcrt* K76T mutation. We also detected two mutant haplotypes of *pfcrt* gene (IET and MNT), with the haplotype IET (54.7%) more prevalent than the other, and significantly higher than the average level (30%) in Africa in a recent study [33]. CQ was the first-line antimalarial drug in Africa during 1998–2008. Although previous studies have illustrated partial recovery of CQ sensitivity after CQ withdrawal [34, 35], a report has pointed out that the return of CQ-susceptible malaria to the entire African region is not a synchronous process [25]. The high prevalence of *pfcrt* K76T mutation in this study may be related to the special population (imported malaria) and the limited sample size. Here we also found a significant difference in the prevalence of IET haplotype between East Africa (23%) and West Africa (65%). The CQ resistance was initially identified in East Africa in the late 1970s, and countries in this area were the first to change their front-line treatments from CQ to other antimalarial drugs [36, 37]. In this study, only one case was detected the MNT haplotype, which is consistent with the fact that MNT is more common in South America and some Asian countries, while rarely reported in Africa [38].

The *pfmdr1* with the N86Y and D1246Y mutation can confer resistance to a wide range of first-line antimalarial drugs in vivo and in vitro such as CQ and AS-AQ, especially for the N86Y [39]. The Y184F mutation is more popular in Africa, which has only a weak association with antimalarial effectiveness in vivo and in vitro depending on the status of residue 86 [33, 40]. In this study, we detected 3 SNPs (N86Y, Y184F, and D1246Y) in imported falciparum malaria cases, of which Y184F (72.2%) was the most prevalent, while N86Y and D1246Y being detected in only 22.2 and 5.6%. Currently, many studies have shown that N86Y increases parasite susceptibility to LMF, MQ, and DHA, and augments resistance to md-AQ and CQ [41, 42]. When parasites are engineered to express wild-type N86, they demonstrate a higher drug IC_50_ (inhibitory concentration 50%) value for MQ and LMF, compared with the mutant N86Y residue [33]. Consistently, other studies have reported that the N86 allele predominates in recurrent infections following AL treatment [17, 19]. And this has been verified by a meta-analysis of 31 clinical trials [40]. Therefore, the low prevalence of N86Y in this study may indicate a decreased parasite susceptibility to AL, MQ, and DHA. The high frequency of Y184F shows an increased resistance to PPQ when paired with N86. Among the 5 mutation haplotypes, the NFD haplotype had the highest prevalence of 53.7%. A previous study has shown a significant resistance to PPQ in NFD parasites with IET background in Southeast Asia [33, 40]. Furthermore, the parasites expressing the combination haplotype NFDIET were predominant in this study. Therefore, these cases should be monitored when AL and DHA-PPQ are used as the first-line anti-malaria drugs.

To date, more than 200 SNPs have been detected in the *K13*-*propeller* gene of *P. falciparum* isolates worldwide, of which 5 SNPs are validated to be resistance mutations by in vitro and in vivo data and 26 SNPs are candidate resistance mutations correlated with delayed parasite clearance [22]. In this study, we identified 5 SNPs in 7 isolates, of which 3 were nonsynonymous mutations and 2 were synonymous mutations. Among the 3 nonsynonymous mutations, only a candidate K13 resistance mutation, C469F, was identified for the first time in a *P. falciparum* isolate from Democratic Republic of the Congo with the prevalence of 2.0%. C469F and C469Y are two types of mutation only found in Southeast Asia with low prevalence that are candidate mutations to be correlated with delayed parasite clearance [22, 43]. Therefore, the first detection of C469F mutation in imported cases from Africa raises concerns about the emergence of artemisinin resistance in Africa. S459T was identified in a *P. falciparum* isolate of Tanzania with the prevalence of 2.0%. S459L has also been reported in both Sub-Saharan Africa and Southeast Asia [23]. Two synonymous mutations R471R and V589V were identified with prevalence of 6.0 and 2.0% respectively. The R471R mutation has also been identified in *P. falciparum* isolates from Angola by Yang et al. [44]. The low-frequency mutations in *K13 propeller* gene have been detected widely in many studies in Africa. However, the candidate resistance mutation found for the first time increases the risk of emergence and dissemination of artemisinin resistance in Africa.

## Conclusions

The present study demonstrates the current status of anti-malarial drug resistance in imported falciparum malaria cases in Guizhou Province, China. CQ resistance pressure still exists. AL and DHA-PPQ are not recommended for use in imported falciparum malaria cases in Guizhou Province. There is a potential risk of artemisinin resistance in Africa since a candidate K13 resistance mutation has been found. Based on the current results, it is important and imperative to continue a molecular surveillance of anti-malarial drugs for imported malaria cases, which will be helpful to rationalize drug guidance for local authorities in China.

## Supplementary information

**Additional file 1: Figure S1.** Nucleotide sequences alignment of *pfcrt* K76T of imported *Plasmodim falciparum* isolates from Africa in Guizhou Province. The detected locus is highlighted in red, and the sequence polymorphism is marked in yellow.

**Additional file 2: Figure S2.** Nucleotide sequences alignment of *pfmdr1* N86Y, Y184F of imported *Plasmodim falciparum* isolates from Africa in Guizhou Province. The detected locus is highlighted in red, and the sequence polymorphism is marked in yellow.

**Additional file 3: Figure S3.** Nucleotide sequences alignment of *pfmdr1* D1246Y of imported *Plasmodim falciparum* isolates from Africa in Guizhou Province. The detected locus is highlighted in red, and the sequence polymorphism is marked in yellow.

## Data Availability

The sequence alignment information is provided in Supplementary figures. The datasets generated during the study are not publicly available due to the personal information but are available from the corresponding author on reasonable request.

## Abbreviations

ACT: Artemisinin-based combination therapy
WHO: World Health Organization
AS-AQ: Artesunate/amodiaquine
AL: Artemether/lumefantrine
DHA-PPQ: Dihydroartemisinin/piperaquine
SNPs: Single nucleotide polymorphisms
