# Supplementary material for: Polymorphisms of pfcrt, pfmdr1, and K13-propeller genes in imported falciparum malaria isolates from Africa in Guizhou province, China
Source: BMC Infect Dis. 2020 Jul 16;20:513. doi: 10.1186/s12879-020-05228-8 (PMC7364468; doi:10.1186/s12879-020-05228-8)
Supplement: Supplementary file 1 — Additional file 1: Figure S1. Nucleotide sequences alignment of pfcrt K76T of imported Plasmodim falciparum isolates from Africa in Guizhou Province. The detected locus is highlighted in red, and the sequence polymorphism is marked in yellow. [file 12879_2020_5228_MOESM1_ESM.pdf]

Alignment Report of 'PF CRT.meg' - ClustalW (Slow/Accurate, IUB) : Monday, June 22, 2020 10:22 PM

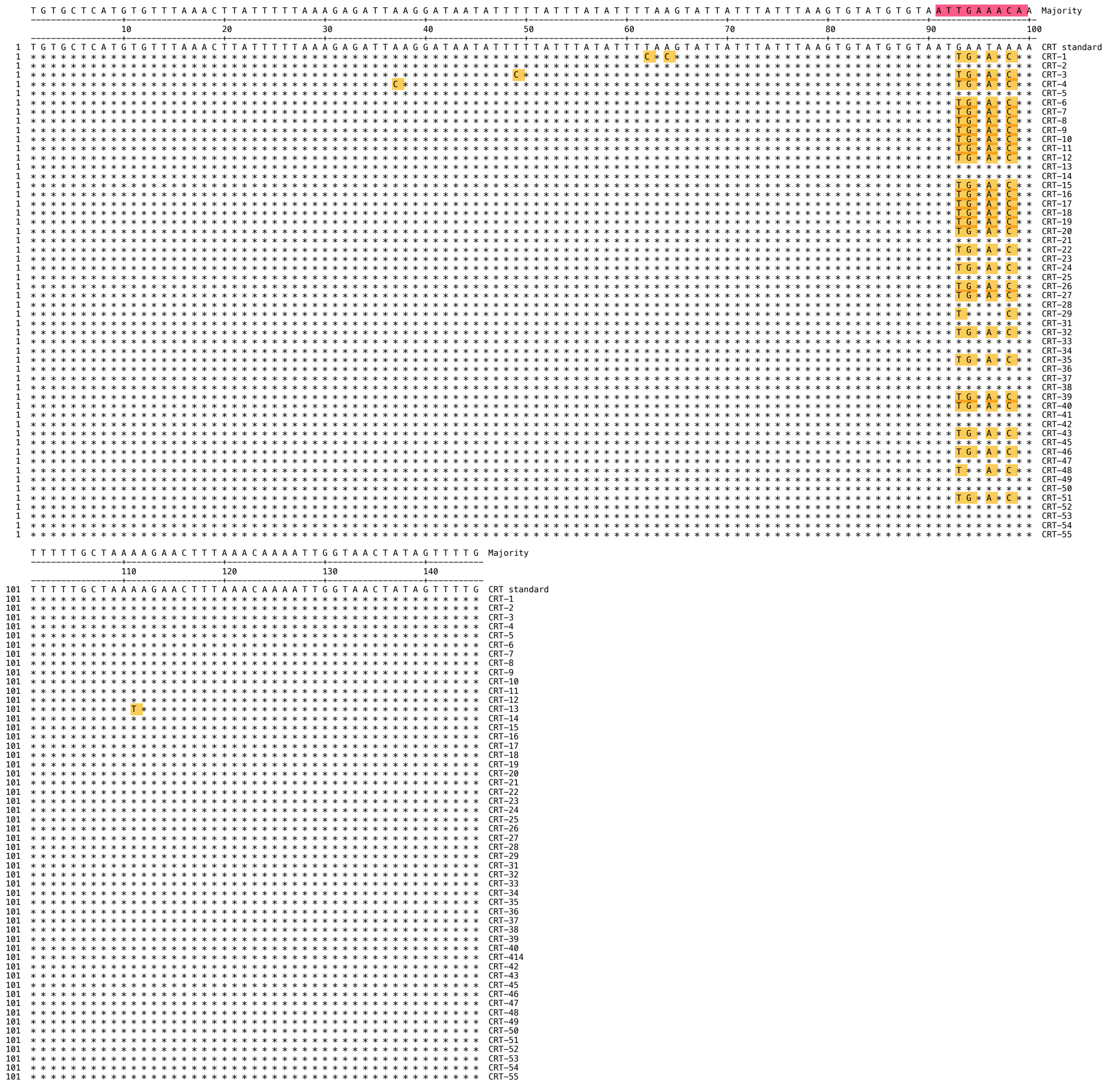

Fig.S1 Nucleotide sequences alignment of pfcrt K76T of imported Plasmodim falciparum isolates from Africa in Guizhou Province. The detected locus is highlighted in red, and the sequence polymorphism is marked in yellow.
