# Supplementary figures and images for: Polymorphisms of pfcrt, pfmdr1, and K13-propeller genes in imported falciparum malaria isolates from Africa in Guizhou province, China
Source: BMC Infect Dis. 2020 Jul 16;20:513. doi: 10.1186/s12879-020-05228-8 (PMC7364468; doi:10.1186/s12879-020-05228-8)

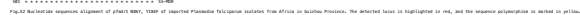

Supplement: Supplementary file 2 — Additional file 2: Figure S2. Nucleotide sequences alignment of pfmdr1 N86Y, Y184F of imported Plasmodim falciparum isolates from Africa in Guizhou Province. The detected locus is highlighted in red, and the sequence polymorphism is marked in yellow. [file 12879_2020_5228_MOESM2_ESM.pdf]

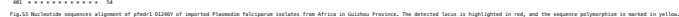

Supplement: Supplementary file 3 — Additional file 3: Figure S3. Nucleotide sequences alignment of pfmdr1 D1246Y of imported Plasmodim falciparum isolates from Africa in Guizhou Province. The detected locus is highlighted in red, and the sequence polymorphism is marked in yellow. [file 12879_2020_5228_MOESM3_ESM.pdf]
